# Impact of genetic polymorphism on perioperative bleeding in adult patients undergoing liver transplantation

**DOI:** 10.1186/2197-425X-3-S1-A232

**Published:** 2015-10-01

**Authors:** MH Starczewska, D Giercuszkiewicz, J Piwowarska, G Kostrzewa, G Niewinski, M Krawczyk, A Kanski

**Affiliations:** II Department of Anaesthesia and Intensive Care Medicine, Medical University of Warsaw, Warsaw, Poland; Department of Forensic Medicine, Medical University of Warsaw, Warsaw, Poland; Department of Medical Genetics, Medical University of Warsaw, Warsaw, Poland; Department of General, Transplant and Liver Surgery, Medical University of Warsaw, Warsaw, Poland

## Intr

Perioperative bleeding remains one of the major causes of increased mortality and morbidity in patients (pts) undergoing liver transplantation (LT). Only few studies have examined the association between genetic polymorphisms and increased blood loss in the population of surgical pts. There are no data regarding this issue neither in liver nor in any other solid organ transplant recipients.

## Objectives

To evaluate the risk exerted by each of the following polymorphisms on perioperative blood loss in adult pts undergoing LT from cadaveric donors: platelet glycoprotein (GP) IaIIa -52 C/T and 807 C/T, GP Iba 524 C/T, tissue factor (TF) -603 A/G and tissue factor pathway inhibitor (TFPI) -399 C/T.

## Methods

We have prospectively enrolled 78 adult pts undergoing cadaveric LT in Central Teaching Hospital of the Medical University of Warsaw. Pts were recruited from August 2012 until March 2014. Written informed consent was obtained from all pts. All subjects were tested for the above polymorphisms. For each polymorphism an allele carrying increased risk for perioperative bleeding (pro-haemorrhagic) was hypothesized based on available data on their physiological role: *allele T* (GP IaIIa -52C/T), *allele C* (GP IaIIa 807 C/T), *allele C* (GP Iba 524 C/T), *allele A* (TF -603 A/G) and *allele C* (TFPI -399 C/T). The primary end point was number of units of red blood cells (RBC), fresh frozen plasma (FFP), platelet concentrates (PLT) and cryoprecipitate transfused during LT.

## Results

The frequency of pro-haemorrhagic alleles in the study population varied from 0.51 to 0.90. The percentage of pts being homozygotes for the pro-haemorrhagic alleles was 24% for GP IaIIa -52 C/T, 28% for TF -603 A/G, 50% for GP IaIIa 807 C/T, 64% for TFPI -399 C/T, and 81% for GP Iba 524 C/T. Multivariate linear regression analysis showed that TF -603 AA genotype was independently associated with increased amount of RBC transfused intraoperatively (β = 0.221; p < 0.05). There was no relation between the amount of RBC transfused and other polymorphisms analyzed. GP IaIIa -52 TT genotype was independent predictor of the amount of FFP (β = -0.197; p < 0.05) and PLT (β = -0.222; p < 0.05) used intraoperatively. No association was found for other polymorphisms. None of the analysed genotypes have significantly affected the amount of cryoprecipitate transfused during LT. Genetic factors were explaining 4%, 5% and 3% of the variablity of the amount of respectively RBC, FFP and PLT transfused intraoperatively.

## Conclusions

This is the first study to demonstrate that polymorphisms of TF -603 A/G and GP IaIIa -52 C/T are independent predictors of perioperative blood loss in adult pts undergoing LT. Surprisingly, for GP IaIIa -52 C/T an allele hypothesized to be associated with increased blood loss turned to be a protective factor. Therefore further studies are required to explain mechanistic link of this association.

